# Nanoparticle based insulin delivery system: the next generation efficient therapy for Type 1 diabetes

**DOI:** 10.1186/s12951-015-0136-y

**Published:** 2015-10-24

**Authors:** Garima Sharma, Ashish Ranjan Sharma, Ju-Suk Nam, George Priya C. Doss, Sang-Soo Lee, Chiranjib Chakraborty

**Affiliations:** Institute For Skeletal Aging, Hallym University-Chuncheon Sacred Heart Hospital, Chuncheon, 200704 Korea; Amity Institute of Nanotechnology, Amity University, Noida, Uttar Pradesh India; Medical Biotechnology Division, School of Biosciences and Technology, VIT University, Vellore, 632014 Tamil Nadu India; Department of Bio-informatics, School of Computer and Information Sciences, Galgotias University, Greater Noida, India

**Keywords:** Nanocarrier, Insulin delivery, Type-1 diabetes mellitus (T1DM), Oral drug delivery, Pulmonary drug delivery

## Abstract

Diabetic cases have increased rapidly in recent years throughout the world. Currently, for type-1 diabetes mellitus (T1DM), multiple daily insulin (MDI) injections is the most popular treatment throughout the world. At this juncture, researchers are trying to develop different insulin delivery systems, especially through oral and pulmonary route using nanocarrier based delivery system. This next generation efficient therapy for T1DM may help to improve the quality of life of diabetic patients who routinely employ insulin by the subcutaneous route. In this paper, we have depicted various next generation nanocarrier based insulin delivery systems such as chitosan-insulin nanoparticles, PLGA-insulin nanoparticles, dextran-insulin nanoparticles, polyalkylcyanoacrylated-insulin nanoparticles and solid lipid-insulin nanoparticles. Modulation of these insulin nanocarriers may lead to successful oral or pulmonary insulin nanoformulations in future clinical settings. Therefore, applications and limitations of these nanoparticles in delivering insulin to the targeted site have been thoroughly discussed.

## Background

Diabetes mellitus (hyperglycemia), a metabolic disorder, is caused either due to lower insulin secretion by the cells or due to lower binding efficiency of insulin on their cell surface receptors resulting in high blood glucose level. According to the survey in low- and middle-income countries there are 366 million people living with diabetes and the count is expected to rise to 552 million by 2030 [1]. Especially in the developing countries, diabetes has increased rapidly during the last decade. In 21st century, this diseases have the possibility to become a new epidemic in the Middle East, Sub-Saharan Africa, Latin America, India, and the rest of Asia [2]. Symptoms of diabetes include excessive weight loss, polyuria, polydipsia and polyphagia [3]. Diabetes has been categorized as Type 1 and Type 2. Type 1 diabetes is insulin dependent condition, characterized by deficiency of insulin due to destruction of insulin-producing beta cells of islets of Langerhans by autoimmune system in pancreas. While, type 2 diabetes is distinguished as disorders of both insulin resistance and secretion due to defects in insulin receptor on cell membranes [4]. Besides these types of diabetes, gestational diabetes has also been reported in pregnant women. During pregnancy, abnormal hormonal production leads to woman’s sensitivity to insulin resulting in high blood sugar levels [5].

Treatment of diabetes need constant monitoring of blood glucose level, regulating it through modified dietary sugar intake, physical exercise and insulin therapy (subcutaneous administration) to attain normoglycemia [6]. Disadvantages of subcutaneous administration of insulin are hypoglycemia [7], peripheral hyperinsulinemia [8], lipoatrophy, lipohyperatrophy [9], obesity due to intensive therapy [10], insulin neuropathy and insulin presbyopia. Current dosage of injectable insulin, required to maintain acceptable serum glucose level, comprise of up to four subcutaneous injections per day [11] which can cause psychological stress leading to poor patient compliance. Thus, focusing on the alternative route of administration (oral or pulmonary) or reducing the injection doses are beneficial to reduce the inconvenience and drawbacks associated with this conventional method [12–15]. Furthermore, orally delivered insulin reaches systemic circulation after passing through liver similar to physiological insulin secretion while injected insulin may result in peripheral hyperinsulinemia and associated complications. However, major obstructions in developing oral or pulmonary insulin formulations are either enzymatic barriers or physical barriers (i.e. intestinal epithelium), which oral insulin has to overcome [11, 16]. Insulin, 51 amino acid protein, can get deteriorated by gastric pH and intestinal enzymes, and even intestinal epithelial cell membranes serve as absorption barrier for intact peptide structure resulting in less than 1 % bioavailabity of total insulin taken orally [17]. Taken together, restrictions like; fragile nature and short half-lives of proteins may serve as extra barriers in the formulation of oral dosage forms. In this context, over past few decades attempts have been made to develop suitable alternative formulations. Some of the methods include the use of permeation enhancers [18, 19]; protease inhibitors [20, 21], hydrogels [22, 23], and protein–ligand conjugates [24, 25]. Although, significant advancement has been made worldwide in attaining the general objective for a convenient and equally effective oral insulin delivery [15], still sufficient commercial development has not been achieved. As a solution to these challenges, nanocarriers have been considered as the best suited vehicle for oral delivery of insulin [26, 27]. Various nanocarriers, like polymeric or micelles, have granted a promising advancement to acquire desirable biopharmaceutical and pharmacokinetic properties for insulin. Therefore, in this review we have tried to highlight several nanocarrier formulations for insulin delivery related to chitosan coated nanoparticles, PLGA-insulin nanoparticles, dextran-insulin nanoparticles, PACA-insulin nanoparticles and solid lipid-insulin nanoparticles. Moreover, limitations associated with these nanocarriers for insulin delivery has also been discussed.

## Roles and possible mechanisms of nanocarriers in oral drug delivery system

**Fig. 1:**
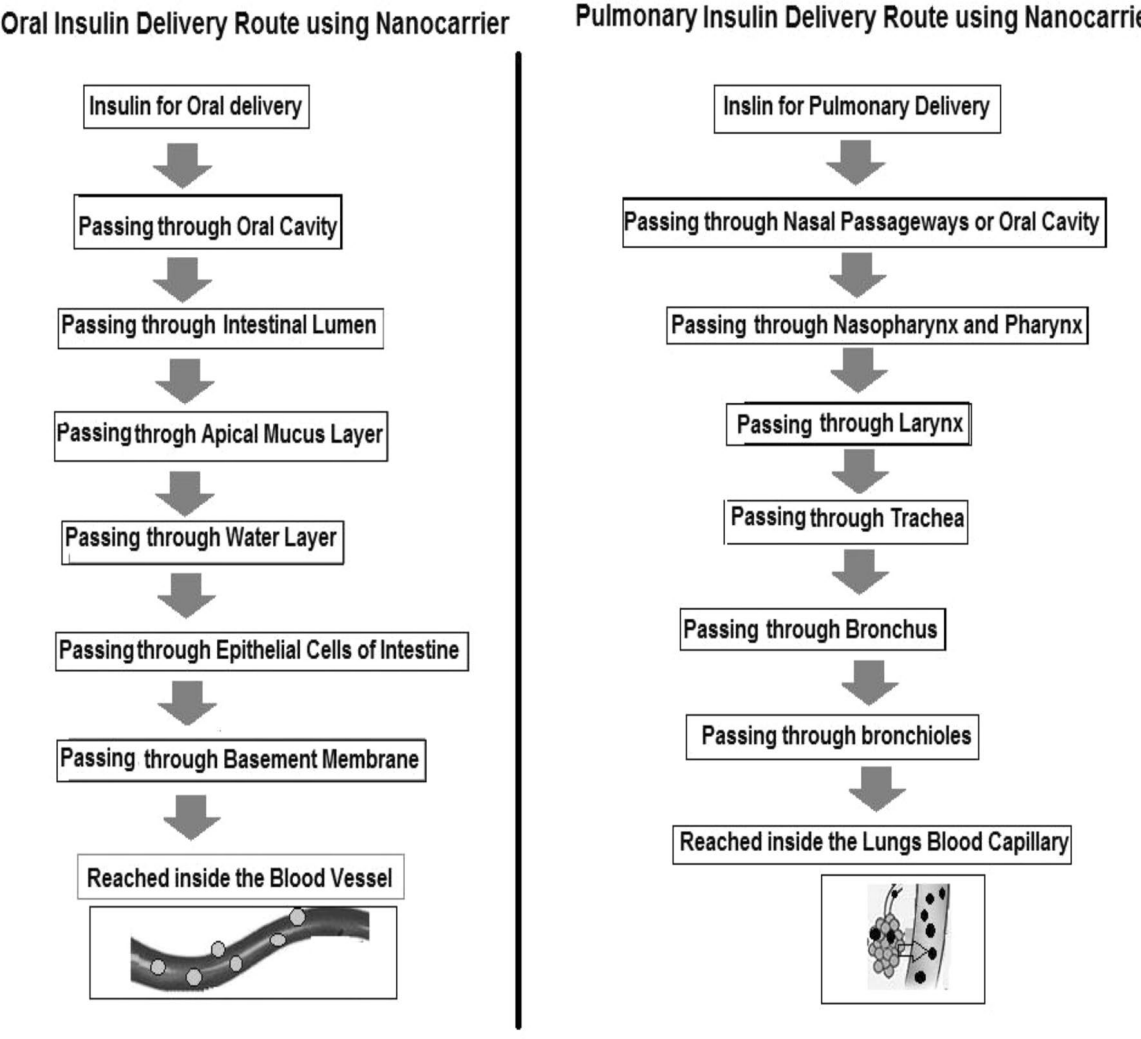
Major two routes of nanocarrier based insulin delivery

The bioavailability of orally delivered drugs is influenced by the physico-chemical properties of the drugs (i.e. solubility, pKa, size, etc.). The absorption of drugs and particles in gastrointestinal tract (GIT) occurs through various sites depending upon their size. Particles with 1 µm diameter are absorbed via phagocytosis by intestinal macrophages while particles <10 µm in diameter are transported through peyer’s patches (lymphatic islands present on GIT). Nanoparticles (<200 nm) are absorbed through endocytosis by enterocytes [28]. The efflux transporters such as P-glycoprotein (Pgp) and enzymes, expressed on enterocytes surface, also render the low systemic bioavailability of drugs affecting the absorption and excretion of drugs. [29]. Nanotechnology reveals the application of size scale complex systems in various fields due to their unique properties [30, 31]. One of the extensively studied areas of nanotechnology is delivering systems for the active ingredient of the medicine. Effective nanomedicine must be stable, biodegradable, non-toxic, non-inflammatory, non-thrombogenic, nonimmunogenic and should escape by reticuloendothelial system [32, 33]. Moreover, nanomedicine should be applicable to different molecules such as small drugs, proteins, vaccines or nucleic acids [34]. It has been proved experimentally that, for therapeutic and imaging applications, nanoparticles may range from 2 to 1000 nm [35]. Additionally, nanotechnology offers the wide range of advantages to the drug delivery field including oral drug delivery in particular, i.e., increase efficacy, tolerability, specificity and therapeutic index of analogous drugs [36]. Furthermore, for oral delivery of drugs nanotechnology may assist in the delivery of poorly water-soluble drugs, transcytosis of drugs across the tight intestinal barrier, targeting of drugs to the specific part of the gastrointestinal tract and in the intracellular and transcellular delivery of bulky macromolecules [37]. Also, to facilitate the oral absorption of peptides and proteins, nanocarriers can be modified with specific ligands and targeted to the receptors on epithelial cell surface [22, 38–41]. Among various limitations of oral delivery of certain drugs is their poor absorption from the GIT. Such limitations can be overcome by the use of bioadhesive polymers which can facilitate the adhesion of nanocarrier to the mucosal epithelial membrane and can assist in nanoparticle uptake [42]. Other than the oral delivery of drugs using nanocarriers, pulmonary means of delivery is also an efficient route (Fig. 1).

The use of biodegradable polymeric nanoparticles have evolved as a better alternative for oral/pulmonary delivery of proteins and peptide drugs [43]. Furthermore, the stability and functional abilities of the nanoparticles can be modulated by some of the pharmaceutically accepted excipients able to regulate pH responsivity and Pgp effect e.g. cyclodextrin, chitosan, PLGA, TPGS/Vitamin E TPGS, etc. [44]. Lowman et al. (1999) formulated pH sensitive nanocarriers to overcome the limitations of oral insulin delivery and observed decrease in blood glucose level for longer time (8 h) in diabetic rats at a dose of 25 IU/kg of loaded insulin [17]. In addition, the controlled release of encapsulated insulin and its enhances uptake and bioavailability can also be modulated by the use of various combinations of polymers and targeted molecules [34, 45]. Some of the pH sensitive biodegradable polymers explored so far are PMAA [46], HPMCP (HP55) [47], dextran sulphate [48], alginate [48], PGA [49] etc.

## Nanocarriers based insulin delivery

**Fig. 2:**
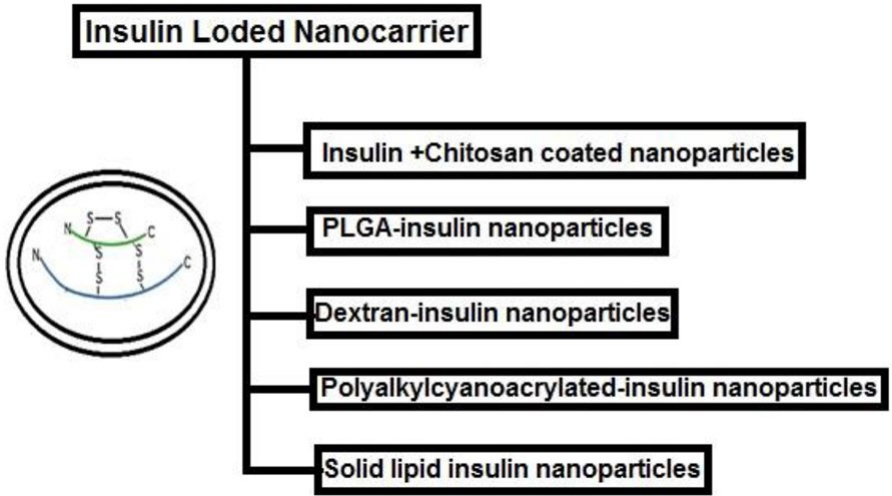
Different types of insulin loaded nanoparticle based delivery system

Due to the drawbacks of conventional injectable insulin, drugs have been modified through nanocarriers with targeting ligands for their selective and targeted delivery meant for oral and pulmonary delivery [22, 41]. Different nanoparticles developed to form stable and efficient insulin delivery system (Fig. 2) are discussed below.

### Chitosan coated nanoparticles

Chitosan is a linear polysaccharide, composed of β-(1-4)-linked d-glucosamine and *N*-acetyl-d-glucosamine. The hydrophilic drugs, like insulin, cannot diffuse across epithelial cells as the intestinal epithelium is a major barrier for their absorption. So, it is difficult for them to enter the bloodstream [16]. Therefore, transport of hydrophilic drugs via paracellular pathways has been studied in detail [27]. Nevertheless, the presence of tight junctions at the luminal aspect of adjacent epithelial cells restricts the transport of hydrophilic drug through paracellular pathway [50–53]. Chitosan is a nontoxic cationic polysaccharide which has been used as the permeation enhancer for the absorption of hydrophilic molecules [54–57]. Chitosan adheres to the mucosal surface and opens up the tight junction between epithelial cells [58–60]. The expression of Claudin-4, a transmembrane protein responsible for tight junction integrity, is Chitosan mediated [61]. Thus, advances in developing stable and efficient chitosan-based particulate insulin delivery system have been examined [60, 62, 63]. Enhanced permeation of chitosan-insulin nanoparticles, synthesized by ionic gelation method using tripolyphosphate sodium (TPP) or poly(acrylic acid) (PAA), has been reported [64]. Further, Lin et al. (2007), showed that chitosan-insulin nanoparticles prolong the residence time of insulin in the small intestine and enhance the permeation of insulin via paracellular pathway to the blood stream. TEM micrographs showed mechanistic details that the chitosan can reversible open tight junctions between Caco-2 cells which increased paracellular permeability [65]. Chitosan-insulin nanoparticles infiltrate into the mucus layer and transiently open the tight junctions located between epithelial cells. In turn, these nanoparticles have become unstable due to pH sensitivity and degrade releasing encapsulated insulin [66]. Chitosan-insulin nanoparticles have also been studied for their significant adsorption characteristics via nasal route of administration [67, 68]. These nanoparticles were synthesized by ionotropic gelation of chitosan and insulin loading was mediated by ionic interaction mechanism. Polyelectrolyte complexation method was also used for insulin loaded chitosan/alginate nanoparticles and showed their internalization through intestinal mucosa [69–71]. Jelvehgari et al., used complex coacervation method for nanoparticles formation of 199 nm diameter using Eudragit L100-55 and chitosan of various molecular weights with 3.38 % entrapment and 30.56 % insulin loading efficiency [72]. The polyelectrolyte complexes of chitosan and insulin gets easily dissociated in acidic medium of the stomach and released insulin resulted in low pharmacological availability due to degradation by enzymatic activity in the GIT [64]. In order to modulate the rate of insulin delivery from chitosan/alginate nanoparticles, magnetite nanoparticles were synthesized inside chitosan/alginate matrix by coprecipitation method for subcutaneous implant approach [73]. Further, oral insulin formulation was prepared by combining nanoencapsulation and lipid emulsion [74]. These microemulsions prevented insulin from enzymatic degradation and enhanced their bioavailability [75, 76]. Cui et al. [77] improved the oral efficiency of insulin by encapsulating it in the shell of pH sensitive carboxylated chitosan grafted poly(methyl methacrylated) nanoparticles via hydrogen bonding, electrostatic interaction and van der waals forces. These nanoparticles exhibited pH sensitive property with slow release at pH 2.0 and quick release at pH 6.8 and 7.4. Sarmento et al. constructed dextran sulfate and chitosan nanoparticles in varying ratios for entrapping insulin, and showed their efficiency as oral insulin delivery nanoparticulate system [78, 79].

### PLGA-insulin nanoparticles

PLGA is FDA approved biodegradable synthetic polymer used frequently for drug delivery. Using computational analysis, Lassalla et al. showed the presence of hydrophobic and hydrophilic interactions between insulin and PLGA polymer [80]. PLGA nanoparticles were formulated by a modified solvent diffusion technique as model nanocarriers for insulin and potential oral drug delivery system [81–83]. Insulin loaded PLGA (PNP) and PLGA-Hp55 nanoparticles (PHNP) nanoparticles were also investigated as an effective method of reducing serum glucose levels, in vivo. The relative bioavailability of PNP and PHNP compared with subcutaneous (s.c.) injection (1 IU/kg) in diabetic rats observed was 3.68  ±  0.29 and 6.27  ±  0.42 %, respectively [47]. Hp55 was used as a pH sensitive cellulose coating to resist high acidic pH of gastric fluids for longer time simultaneously dissolving in lower acidic pH of small intestine. Double emulsion solvent evaporation method was also used to design PLGA encapsulated insulin nanoparticles and then embedded within PVA hydrogels. This composite system showed a reduction in both the release rate and the total amount of insulin released [84]. Attempts have been made to modify the slight negative surface charge of PLGA by using polycationic polymer, chitosan. Because of the positive surface charge, chitosan reverses the effect of negative charge on PLGA further supporting endocytosis of nanoparticles through their increased interaction with the cell membrane [85]. Previously, chitosan has been known as one of the Pgp modulator which may decrease the Pgp-mediated efflux of drug loaded nanoparticles from the luminal surface of cells [86]. As a result, chitosan modified PLGA nanoparticles exhibited strong bioadhesive potency and increased pharmacological availability with regard to orally delivered insulin [87]. PLGA nanoparticles harbouring insulin-S.O (sodium oleate) complex was prepared via an emulsion solvent diffusion method and was evaluated for their pharmacological effects via oral administration to diabetic rats [88]. It was seen that, after 12 h of administration, plasma glucose level was reduced to 23.85 % from the initial level, and this conditioned was maintained till 24 h. S.O is an anionic surfactant which forms an ionic complex with positively charged insulin at suitable pH and improves the apparent liposolubility of insulin. Additionally, the structure of polymers used to fabricate drug loaded nanoparticles can also impact their functional properties. Thus, in another method bovine insulin was entrapped in linear PLGA-PEG, star-branched β-cyclodextrin-PLGA (β-CD-PLGA), and glucose-PLGA (Glu-PLGA) copolymeric nanoparticles using double emulsion method to enhance the complexation between insulin and polymers for their sustained release for 24 h [89]. Such kind of approach can provide single oral dose which could eliminate the need for repeated insulin doses till 24 h. In a similar study, folate (FA) coupled PEG-PLGA nanoparticles were used to encapsulate insulin by solvent evaporation method and showed that once-daily administration would be sufficient to control diabetes for at least 24 h [90].

### Dextran-insulin nanoparticles

Earlier studies suggest that the best way to treat diabetes is to provide exogenous insulin level according to the blood glucose level of the patient [91]. Although the methods described above enhance insulin delivery process, still their release mechanism is not proportional to the required physiological blood sugar concentration. To achieve the goal of glucose responsive release of insulin, the researchers have focused on novel nanomaterials. Among these approaches, competitive binding is the most acceptable one [92–95]. Synthesizing nanoparticles with such glucose responsive materials would carry the advantages of nanosized particles as well as glucose response dependent release of insulin in the body.

Zion et al. (2003), synthesized a novel reverse microemulsion (RM) mediated glucose-responsive dextran, poly(α-1,6 glucose), nanoparticles which was physically crosslinked with the tetrafunctional glucose-binding protein, Concanavalin A (Con A), for controlled insulin delivery [96]. Upon contact with free glucose, Con A releases polymeric glucose and further binds to free glucose, leading to disintegration of hydrogel. As discussed above, insulin is marginally stable and can easily break up during their formulation as drugs [97]. Therefore, in order to achieve stable insulin formulation, aqueous insulin encapsulating nanoparticle delivery system was developed. This method utilized oppositely charged dextran sulfate (DS) and polyethylenimine (PEI) along with zinc as a stabilizer and was tested for insulin stability. However, this system showed no significant conformational changes in encapsulated insulin as compared to free insulin [98]. Recently, for oral delivery of peptides the use of some natural uptake processes of the intestine like vitamin B12 (VB12) transport system has also been highlighted which utilizes VB12-IF-IFR (intrinsic factor receptor) mediated endocytosis through intestinal ileocytes for targeting systemic circulation [99–101].

VB12–dextran NPs conjugates, chemically coupling insulin, acting as an oral delivery system has also been attempted to protect insulin against gut proteases and to show a faster release profile [41, 102]. These nanoparticle conjugates were found to be viable carrier for personal insulin delivery to treat diabetes. A multilayered nanoparticle system consisting of mucoadhesive polymers, sodium alginate and dextran sulfate, around calcium was also developed to entrap insulin which enhances the residence time at absorption site. This system was further stabilized by chitosan bound to ploxamer 188 further coated with albumin A to protect insulin from enzymatic degradation. This nanoformulation of insulin exerted an efficient and persistent hypoglycemic effect in diabetic rats [103]. In a similar study, Reis et al., synthesized mucoadhesive, biodegradable, biocompatible and acid protected the sodium alginate and dextran sulfate nanospheres, having insulin in their core. Additionally, these nanospheres were coated with chitosan, BSA and PEG 4000 [104].

### Polyalkylcyanoacrylated-insulin nanoparticles

Initially, PACA were used as a tissue glue [105] in surgery because of their stable and biodegradable character [106]. Recently, it has been utilized in the transportation of insulin through intestinal epithelium polymeric insulin carrier for oral administration [107]. According to MALDI ionization coupled tandem time-of-flight (TOF) mass spectrometry analysis, insulin was not modified during covalent bonding with PACA nanoparticles [108]. Entrapment of insulin in PACA nanoparticles prepared from microemulsions with the different microstructure containing isopropyl myristate, caprylocaproyl macro golglycerides, polyglyceryl oleate and insulin solution were investigated for in vitro release and bioactivity [109]. Moreover, insulin-loaded polybutylcyanoacrylate nanoparticles (IPN) were also tried for the hypoglycemic effect upon oral administration to streptozotocin (STZ) induced diabetic rats in an oily medium (soybean oil containing 0.5 % (v/v) Tween-20 and 5 % (v/v) Vitamin E). It was concluded that IPN can serve as an effective and stable delivery system for oral insulin [110].

### Solid lipid insulin nanoparticles

As an alternative to polymeric nanoparticles, solid lipid nanoparticles (SLN) were developed for drug delivery nanoparticulate system [111]. SLN is sub micron, around 50–1000 nm in diameter, colloidal carriers made up of lipids which are solid at room temperature. SLN can be dispersed in water or surfactant solution [112]. Advantages of SLN as nanoparticle carrier systems are biodegradability [111], increased bioavailability, extended blood residence time [113], high tolerability [114] and easy large scale commercial production [113, 115, 116]. Moreover, SLN can be taken up by the lymphoid tissues in the peyer’s patches. Oral administration of lectin modified SLNs with loaded insulin demonstrated declined enzymatic degradation and enhanced oral absorption [117]. It is well known that lectins consist of a diverse class of proteins having the capability to bind specific carbohydrates. Since, many proteins and lipids of GIT membrane are glycosylated, these lectins render a suitable alternative for recognition and enhanced uptake of drug loaded nanocarriers by intestinal mucosal membrane. In vivo hypoglycemic effect of SLN containing insulin, synthesized by solvent emulsification evaporation method, was studied for 24 h, and it was seen that SLN can encourage the oral absorption of insulin. This method was based on a w/o/w emulsion technique [115]. Insulin mixed micelle loaded SLNs was prepared with reverse micelle double emulsion method using the mixture of stearic acid and palmitic acid. The liposolubility of insulin was improved by using sodium cholate and soybean phosphatidylcholine. This insulin delivery system had an excellent long term stability at 4 °C [118]. Octaarginine is an arginine rich derivative which is known as cell penetrating peptide assisting in uptake of various drug carriers. Zhang et al. 2009 attempted octaarginine modified SLN as oral insulin delivery system [119]. Internalization of above mentioned insulin-SLN by Caco-2 cells was increased by 18.44 folds as compared to insulin solution [120]. Furthermore, researches focused on coating SLN with chitosan. Mainly due to the fact that non-coated SLN were shown to be uptaken by RAW 264.7 cell lines, whereas chitosan coated SLN were not internalized by this macrophage cell line. This may be due the fact that the addition of stealth layer on SLN by chitosan may enable SLN to escape phagocytosis [121]. Another such approach was carried out to produce insulin entrapped chitosan-coated Witepsol 85E SLNs. At first, solvent emulsification–evaporation method based on a water/oil/water double emulsion method was used to produce SLN, followed by chitosan coating on SLN surface. This work too showed enhanced permeation of chitosan coated SLN in comparison to noncoated SLN [122]. Studies were also performed to find out suitable lipid materials to synthesize insulin loaded SLN, and it was seen that glyceryl palmitostearate was the best suited lipid in terms of hydrophobicity, lower burst release and high pharmacological availability [123]. Besides oral delivery, SLNs was also used for pulmonary delivery of insulin. In this method, both cationic and anionic insulin-SLN nanoparticles were prepared and were then allowed to self assembled into flocculates due to electrostatic interactions. Finally, the flocculates were lyophilized to form dry powder for pulmonary administration [124].

### Other targeted nanoparticles encapsulating insulin

Targeted ligand modified nanocarriers were proposed earlier to facilitate the oral absorption of proteins and peptides [38]. Some of the reported targeting agents to enterocytes or M cells are lectins, transferrin and vitamin B12 [22, 39–41]. However, the targeting effect of these ligands can be hindered by the presence of the mucus layer on the epithelium [125, 126]. Therefore, more efficient targeting and highly specific ligands need to be explored which can overcome the mucus barrier on epithelium. Lately, a peptide was identified which have an affinity with goblet cells. Goblet cells consist of the second largest population of cells in intestinal epithelia. This peptide, CSKSSDYQC, was identified from phage-peptide library using in vivo phage display method [127]. Reports suggested enhanced uptake of CSKSSDYQC modified insulin containing chitosan nanoparticles in villi and increased permeation of insulin across the goblet cell-like HT29-MTX cells through clathrin and caveolae mediated endocytosis [26]. Recently, transportation of insulin loaded nanoparticles to neonatal Fc receptor (FcRn) across intestinal epithelium was studied [128]. FcRn are expressed on epithelial cells and assists in IgG transport through them by binding to Fc region of the antibodies in a pH dependent manner. Therefore, the transportation of various nanoparticles to FcRn can be facilitated by the use of Fc region of IgG as a targeting ligand.

## Limitations

Although attempts have been made so far in the development of oral insulin nanoparticulate (Table 1), the formulation and synthesis of more efficient nanoformulation is required for commercial significance. Recently, a number of insulin nanocarriers have undergone clinical trials among which many of them faced failure. Drawbacks associated with them include toxicology, low level of oral bioavailability and elevated intraindividual difference in insulin absorption. In the near future, to develop clinically significant insulin loaded nanocarriers biocompatibility, biodegradability and immunological responses should be considered. As a result, various features have to be taken in account before designing novel insulin nanocarriers. Some of the features are optimum particle size which can interact with the intestinal mucosa, the stability of the nanocarriers in biological fluids after in vivo administration, surface chemical composition, internal chemical composition and use of targeting ligands specific for apical membrane receptors. Additionally, detailed study about distribution kinetics and the interaction of nanocarriers with the mucosal lining of intestinal epithelia is also needed.

**Table 1:** Physicochemical parameters for different types of insulin nanocarrier

Nano-carrier	Method of synthesis	Components	Size (nm)	Zeta potential (mV)	Route of administration of nanocarrier	EE (%)	DL (%)	Dose (I.U./kg) (min/max)	PA (%)	Ref
Chitosan-insulin	Graft polymerization	Carboxylated chitosan + methyl methacrylate	251 to 319	–22 to –26.4	Oral	85	11.9	15 (min) 100 (max)	9.7	[77]
	Polyelectrolyte complexes + oily dispersion system	Chitosan + Oleic acid + Plurol oleique + Labrasol	108	29.97	Oral	30.38 to 78.81	–	50	7	[76]
	Iionotropic pre-gelation + polyelectrolyte complexation	Chitosan + Alginate	748	−5.6	Oral	72.8	9.9	25 (min) 100 (max)	7.1 to 3.4	[79]
	Polyelectrolyte complexation	Chitosan + Dextran sulfate	527	−20.6	Oral	69.3	2.3	50 (min) 100 (max)	5.6 to 3.4	[78]
	Ionotropic gelation	Chitosan + TPP + Poloxamer 188	250 to 400	27.31 to 40.71	Oral	59.6 to 88.6	7.0 to 26.3	7 (min) 21 (max)	14 to 15.3	[60]
	Ionotropic gelation	Chitosan + TPP	269 to 688	21.8 to 49.8	Oral	38.5 to 78.5	60.8	50 (min) 100 (max)	–	[64]
	Ionotropic gelation	Chitosan + TPP	300 to 400	54 to 25	Pulmonary	87.4 to 96.7	19 to 55	5	–	[68]
	Polyelectrolyte complexation	Chitosan	200 to 500	8.8 to 22	–	81.4 to 94.8	90	–	–	[67]
	Ionotropic gelation	Chitosan + Poly(ç-glutamic acid)	110 to 150	−23.7 to 33.4	Oral	56.8	14.9	15 (min) 30 (max)	–	[70]
	Ionotropic complexation + coacervation	Chitosan + Dextran sulfate + Alginate	423 to 850	–	Oral	3 to 94	5 to 13	–	–	[71]
	Coacervation method	Chitosan + Eudragit L100-55	135 to 199	−20.7 to −27.9 mV	Oral	3.38	30.56	–	–	[72]
	W/O/W multiple emulsion + polyelectrolyte cross-linking	Chitosan + Alginate + Calcium chloride + Labrafac CC + Phospholipid + Span 80 + Cremorphor EL	488	−62.25	Oral	47.3	–	25 (min) 50 (max)	8.19 to 7.84	[74]
	Polyelectrolyte complexation	Chitosan + *γ*-PGA	185.1 to 198.4	29.9 to 27.8	Oral	40.1 to 55.1	5.2 to 14.1	30	13.0	[65]
PLGA-Insulin	Reverse micelle–solvent evaporation method	PLGA + Phospholipid + PVA	102 to 428	−12 to −22	Oral	51.5 to 90.4	4	20	7.7	[82]
	Emulsion solvent diffusion method	PLGA + Hp55	169	–	Oral	65.41	3.17	20	6.27	[47]
	W/O/W solvent evaporation technique	PLGA + Chitosan + Pluronic 188	134.4	43.1	Oral	52.76	1.29	15	10.5	[87]
	W/O/W solvent evaporation technique	PLGA + Pluronic 188	121.3	−1.72	Oral	46.87	1.14	15	7.6	[87]
	Hydrophobic ion pairing + emulsion solvent diffusion method	PLGA + Sodium oleate + PVA	161	−33.4	Oral	91.2	–	20	11.5	[88]
	W/O/W double emulsion method	Poly(d,l-lactide-co-glycolide) + poly(ethylene glycol)	120 to 355	–	–	95	–	–	–	[89]
	W/O/W double emulsion method	β-cyclodextrin-PLGA	120 to 355	–	–	95	–	–	–	[89]
	Double-emulsion solvent evaporation method	PLGA + PEG + Folate	∼260	–	Oral	87	∼6.5	50	–	[90]
Dextran-insulin	Emulsion method	Dextrans + Epichlorohydrin + vitamin B(12)	160 to 250	–	Oral	45 to 70	2 to 4	20	11.4 to 26.5	[102]
	Ionotrophic gelation + polyelectrolyte complexation	Dextran + Alginate + Poloxamer + Chitosan + BSA	396	−38.2	Oral	–	–	50	13	[103]
	Nanoemulsion dispersion + triggered instantaneous particle gelation	Dextran + Alginate + Chitosan + PEG + BSA	>1842 (90 %) >812 (50 %)	−7	Oral	85	–	25 (min) 100 (max)	42 to 10	[104]
Polyalkylcyanoacrylate-insulin	Microemulsion	Isopropyl myristate + Labrasol + Plurol Oleique + Ethyl (2) cyanoacrylate	200 to 400	−22 to −11.4	Oral	16 to 32.6	1.82 to 0.62	100	–	[109]
	Microemulsion	Isopropyl myristate + Labrasol + Plurol Oleique + butyl (2) cyanoacrylate	200 to 400	−21.5 to −6.5	Oral	11.5 to 52.3	1.31 to 0.99	100	–	[109]
	–	Polybutylcyanoacrylate + Tween 20	78	–	Oral	–	–	50	15.5	[110]
	–	Polybutylcyanoacrylate + Tween 20 + Soyabean oil + vitamin E	67	–	Oral	–	–	50	22.4	[110]
Solid lipid-insulin	W/O/W double emulsion technique	Cetyl palmitate	361	−3.4	Oral	43	–	50	5	[115]
	–	Lecithin + stearic acid + ploxamer + wheat germ agglutinin-*N*-glutamyl-phosphatidyl-ethanolamine	75.3	−13.11	Oral	17.89 to 23.72	–	50	6.08	[117]
	Reverse micelle-double emulsion	Sodium cholate (SC) + soybean phosphatidylcholine + stearic acid + palmitic acid	114.7	−51.36	–	97.78	18.92	–	–	[118]
	W/O/W emulsion technique	–	2 µ	–	Pulmonary	56.32 to 66.02	–	8	35.62	[124]
	Double emulsion method	Stearic acid/octadecyl alcohol/cetyl palmitate/glyceryl monostearate/glyceryl palmitostearate/glyceryl tripalmitate/glyceryl behenate	213 to 444.8	−9	Oral	–	–	50	2.92 to 4.53	[123]
	Solvent emulsification-evaporation	Witepsol 85E	243	−25	Oral	43.6	2.1	25	8.26	[122]
	Solvent emulsification-evaporation	Witepsol 85E + chitosan	470	34	Oral	52.2	1.4	25	17.7	[122]
Targeted insulin nanoparticle	Ionotropic gelation method	N-trimethyl chitosan chloride + CSKSSDYQC peptide +	342	3	Oral	55.4	–	50	5.66	[26]
	Nanoprecipitation	PLA-PEG + human polyclonal IgG Fc	63	−5.6	Oral	–	0.5	1.1	13.7	[128]

*EE* encapsulation efficiency, *DL* drug loading, *PA* pharmacological availability, *Ref* references

## Conclusion

Presently, nanoparticle based drug delivery system are playing an essential role in the pharmaceutical industry. A new drug delivery system of an existing drug can provide a new marketability which is the important in the economic point of view. The next generation nanoparticles based insulin may be the future medicine for T1DM. In the near future, this nanocarrier based insulin delivery could replace the traditional and most predictable subcutaneous insulin injections. Possibly this next generation nanoparticle mediated insulin may improve efficacy of this medicine and will also help the better quality of the living of T1DM patients.

## Abbreviations

PLGA: Poly(lactic-co-glycolic acid)
PACA: Poly(alkylcyanoacrylate)
TPGS: d-α-tocopheryl polyethylene glycol 1000 succinate
PMAA: Poly(methacrylic acid)
HPMCP: Hydroxypropyl methylcellulose phthalate
PGA: Poly glutamic acid
FDA: Food and drug administration
PVA: Polyvinyl alcohol
PEG: Polyethylene glycol
BSA: Bovine serum albumin
MALDI: Matrix-assisted laser desorption ionization
